# Segmental bioelectrical impedance analysis for Korean older population with cold pattern

**DOI:** 10.3389/fnut.2022.975464

**Published:** 2022-12-01

**Authors:** Dieu Ni Thi Doan, Kahye Kim, Seul Gee Kim, Siwoo Lee, Kun Ho Lee, Jaeuk Kim

**Affiliations:** ^1^Digital Health Research Division, Korea Institute of Oriental Medicine, Daejeon, South Korea; ^2^School of Korean Convergence Medical Science, University of Science and Technology, Daejeon, South Korea; ^3^Gwangju Alzheimer’s Disease and Related Dementias (GARD) Cohort Research Center, Chosun University, Gwangju, South Korea; ^4^Department of Biomedical Science, Chosun University, Gwangju, South Korea; ^5^Dementia Research Group, Korea Brain Research Institute, Daegu, South Korea

**Keywords:** cold pattern recognition, body composition, segmental bioimpedance analysis, sex difference, cellular water disturbance

## Abstract

**Objective:**

This study examined the association of whole-body composition and segmental bioimpedance variables with cold pattern (CP) in different sexes.

**Methods:**

We assigned 667 older individuals to a CP group (*n* = 488) and a non-CP group (*n* = 179) by using an eight-item self-administered questionnaire. Seven body composition variables and three pairs of segmental bioimpedance variables for the upper and lower extremities, which were obtained from a segmental multifrequency bioimpedance analyzer, were employed to investigate their association with CP. Participants’ characteristics were first described. Then we compared the selected body composition and bioimpedance variables between the CP and non-CP groups. Finally, their association with CP was investigated using univariate and multivariate regression analyses. All analyses were performed separately for women and men.

**Results:**

Both women and men exhibited a comparable mean age in the CP and non-CP groups; however, women with CP had significantly lower blood pressures, whereas men with CP showed a higher proportion of osteoarthritis than those without CP. Compared with the non-CP group, individuals with CP exhibited significantly smaller body sizes indicated by shorter height and smaller weight, lower body mass index, and smaller volume-to-body surface area ratio in both sexes. After controlling for age, height, weight, and other covariates, we found significant reductions in body lean mass such as fat-free mass and body cell mass, basal metabolic rate per unit mass, total body water, and intra-to-extracellular water ratio in the CP group. With regard to segmental bioimpedance analysis, the resistance ratios and phase angles in the upper and lower extremities yield significant associations with CP incidence, as demonstrated by the odds ratio (95% confidence interval) of 1.72 (1.16–2.57), 1.69 (1.18–2.48), 0.60 (0.40–0.89), and 0.57 (0.39–0.82), respectively. However, these results did not emerge in men.

**Conclusion:**

Abnormal cellular water distribution and deterioration in body cell mass and/or cell strength are associated with CP prevalence, regardless of age, height, weight. These findings are similar in the upper and lower extremities and are more pronounced in women. The abovementioned patterns may be considered effective indicators for identifying CP in the older adult population.

## Introduction

Cold pattern (CP) is a medical term commonly used to describe cold sensitive symptoms, including aversion to cold, feeling of cold on the extremities or body, feeling comfortable in warm conditions, having a pale face and colorless urine, and relevant characteristics of pulse and tongue (1). These cold hypersensitive symptoms are common in the general population as shown by its prevalence that reaches up to 12%, according to a community-based survey (2) and 60% in the Korean and Japanese populations (3, 4).

From traditional medicine perspective, CP is one of the two fundamental principles that are related to the nature of diseases that manifest from yin-yang transformations (5) and is an important diagnostic factor for several diseases (6). In fact, previous studies reported a number of serious chronic diseases in older adults that have been linked to CP or cold hypersensitivity. For instance, Raynaud’s disease, hypotension, chronic gastritis, and diabetes mellitus are associated with cold hypersensitivity in the hands and feet (7, 8). Women with cold hypersensitivity in their extremities may experience shoulder stiffness, fatigue, low back pain, and headache (9). Additionally, individuals with cold hypersensitivity may encounter many other diseases such as cold-related injuries, rheumatic diseases, nerve injuries, migraines, and vascular diseases, as described in the general population of northern Sweden (10). Noteworthy, individuals with CP have a significantly lower quality of life, as evidenced by significantly lower European Quality of Life Five Dimension indices (11) and poorer sleep quality (12) than those without CP. As it is related to various medical problems, CP has been used to identify pathological patterns by nearly 85% of members of the Association of Korean Medicine when prescribing herbal treatment (13).

**FIGURE 1:**
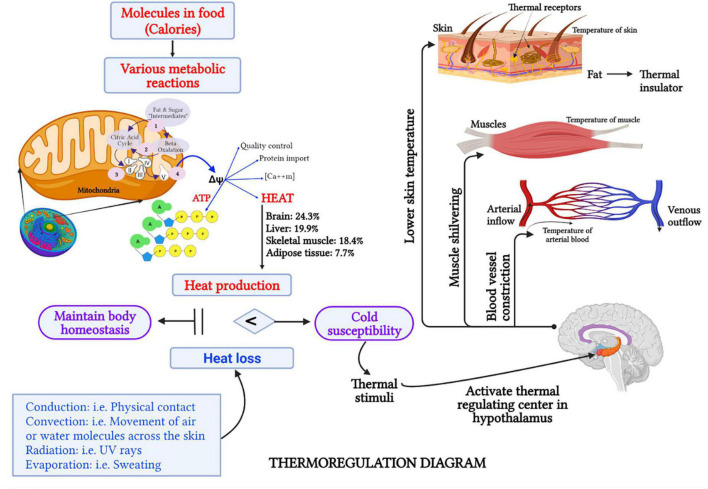
Schematic representation (created with BioRender.com) of the thermoregulation mechanism in the human body. Intracellular mitochondria play a critical role in generating energy (in the form of ATP) and heat, which is sufficient for a cell’s sustenance (52). Healthy cellular mitochondria in the brain, liver, and skeletal muscle mass contribute as much as 63% of the body heat (58). When heat production and heat loss are balanced, human body homeostasis is maintained. However, when heat production is insufficient to compensate heat loss, which occurs *via* conduction, convection, radiation, or evaporation, the human body is likely to be susceptible to cold (85, 86). For a given thermal stimuli, such as cold wind, the thermoreceptors which are free nerve endings in the skin and other organs will send a signal to the hypothalamus to activate the heat-regulating center (87). Consequently, several responses, such as lowering of skin temperature, shivering of skeletal muscle to produce more heat, and vasoconstriction to prevent heat loss, will take place (35). Therefore, individuals with CP usually experience cold sensations in their hands or feet, have a pale face, and seek a warmer environment.

From modern medicine perspective, these symptoms reflect the subjective thermal sensations (e.g., feeling cold), thermoregulation (e.g., pale face due to peripheral vasoconstriction), and behavioral adjustments (e.g., seeking warmth) of a person when subjected to a given climatic stimulus (14). The human body is warmed and powered up during various metabolic reactions, which extract energy from chemical bonds in food molecules to maintain homeostasis (15). Figure 1 briefly explains the thermoregulatory mechanisms in the human body. The metabolic rate is therefore related to thermoregulation, in such a way that enhanced thermogenesis results in an elevated basal metabolic rate (16). Indeed, the metabolic rate at rest is considered a potential parameter that has a relationship with CP as it is negatively associated with CP regardless of age, sex, and fat-free mass (FFM) (17).

In relation to the resting metabolic rate, the other body composition variables such as body weight, body mass index (BMI), skeletal muscle mass, intracellular water (ICM), extracellular water (ECM), and body water ratios have been investigated in relations with CP or cold hypersensitivity (18–20, 21). These previous studies found a significant reduction in BMI and skeletal muscle mass in individuals with CP when compared with the heat pattern or warm sensation group (18, 19). Additionally, Mun and colleagues suggested that sex and BMI might be key features when predicting CP in an aging population (21). Nevertheless, these variables cannot reflect changes at the cellular level such as the cellular hydration, cell integrity, or cell membrane strength, which are essential information of cellular health status.

Evidently, information on body cellular health and its hydration status can be demonstrated based on resistance (R), reactance (Xc), and phase-angle (PA) variables obtained from the bioelectrical impedance analysis (BIA) technique. This technique, which is simple, portable, and inexpensive, can be used effectively to evaluate R, Xc, and PA and estimate body composition variables (22). The BIA assumes that the human body is a homogenous conductive cylinder and its impedance quotient has an empirical relationship with the volume of electrolyte water contained in the FFM (23, 24). R is used as a measure of resistivity and is inversely related to the amount of body water (23). Since low frequency (i.e., 5 kHz) current cannot penetrate the cell membrane while high frequency (i.e., 250 kHz) can pass through the cellular environment, R reflects the ECW volume when measured at a low-frequency and represents the total body fluid when measured at a high-frequency current (24, 25). Accordingly, the ratio between a high- and low-frequency R implies the water ratio between the total body and ECW volumes (26, 27). Xc, the capacitance initiated by the cell membranes, indicates the cell mass, volume, or strength. PA is another parameter that can be calculated based on R and Xc, which represents the phase shift between voltage and current and indicates the ability of the cell membrane to hold charges (23). A reduction in the Xc and PA suggests a lower body cell mass (BCM) or function, whereas a reduction in R implies a relative increase in body fluids and/or FFM with regard to fat components (24, 28).

In addition, segmental BIA evaluates the body segmentally by considering the arms, legs, and trunk as five separate cylinders, and provides R, Xc, and PA measurements for each segment independently (Inbody S10 User’s Manual) (29). Previous studies on various diseases among older people suggested different relations with segmental bioimpedance variables between the upper and lower extremities (30, 31). Therefore, examining the segmental R, Xc, and PA in the upper and lower extremities may provide an insight as to how these values alter in persons with prolonged CP or cold hypersensitivity symptoms, especially in the hands and feet. Furthermore, early detection of CP in individuals based on these variables may enable a prompt and effective treatment to prevent the progression of the related diseases (7–10).

However, to our knowledge, only one relevant study in the community of Jeju Haenyeo has investigated and reported a significant association between the whole-body PA and non-CP with an odds ratio (OR) and 95% confidence interval (CI) of 2.40 (1.16–4.97) (11). Thus, the changes in these body compositions as well as bioimpedance variables were not well-investigated. Consequently, more studies are needed on the association of the body composition and bioimpedance variables with the risk of CP. In this study, we primarily aimed to examine the association between CP and several body composition and bioimpedance variables of the body segments as well as the whole body. Particularly, we explored these associations for different sexes, while considering age and potential comorbidities. We hypothesized that individuals with CP might have lower values in the BCM and/or cell strength as indicated by lower Xc and/or PA variables as well as changes in their cellular hydration status.

## Materials and methods

### Participants

**FIGURE 2:**
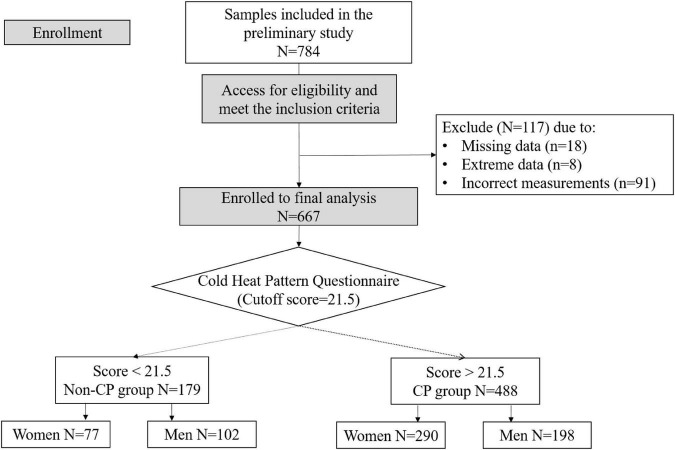
Consolidated standards of reporting trials (CONSORT) diagram illustrating enrollment and exclusion criteria for this study.

A total of 784 participants (age 55–90 years) were recruited at Chonnam University Hospital (Gwangju City, Republic of Korea). Individuals were excluded from the study if they met one of the following criteria: had obtained less than 3 years of education; had a medical history or ongoing acute or chronic illness that interfered with the intended study design such as neurological diseases, infections, or mental health instability; had a cognitive disorder; or had an abnormal skin condition at the location of the measurement probe. In addition to a general medical examination, the participants underwent a precise clinical assessment to obtain information on demographics and medical history. In the data preprocessing, participants who had non-random missing data (*n* = 18) and extreme results (sparse data points above or below three times the interquartile range were considered extreme points) for any parameter (*n* = 8) were excluded from the analysis. According to the principle of multifrequency bioelectrical impedance analysis, correct impedance measurements should have lower values for higher frequencies (i.e., impedance at 1 kHz is greater than impedance at 5 kHz); therefore, observations that did not follow this pattern were excluded (*n* = 91) (31). All of the participants answered the cold heat pattern questionnaire and were accordingly classified into the CP or non-CP groups. Finally, 667 participants, including 488 CP and 179 non-CP individuals, were included in the final analysis (Figure 2). The study protocol was approved by the Institutional Review Board of Chonnam National University Hospital (approval number: CNUH-2019-279). Written informed consent was obtained from all participants. This study was performed in accordance with the principles of the Declaration of Helsinki.

### Body composition, bioimpedance measurement, and data selection

A segmental multi-frequency BIA device was utilized to measure whole-body composition and segmental bioimpedance variables in this study (Inbody S10, South Korea) (29). The device uses a tetrapolar 8-point tactile electrode to compute segmental impedance at six electrical frequencies (1, 5, 50, 250, 500, and 1,000 kHz) and Xc and PA at three frequencies (5, 50, and 250 kHz). These impedance values were then used to estimate seven whole-body composition variables, including fat mass (FM), FFM, BCM, basal metabolic rate per unit mass (BMR_m), ICW, ECW, and ICW_ECW ratio, which were used in this study. According to the formula of Robert (32), BMR_m was calculated as the BMR divided by the fourth root of the body weight (32). Additionally, body volume per unit of surface area was included as a feature of heat diffusion or conduction in considering the body size (33) and was computed using the Sendroy equations (34) (Supplementary Table 1).

For the anthropometric measurements, height (cm) and weight (kg) were automatically measured using the same BIA device to the nearest 1 mm and 100 g, respectively. BMI was then calculated (kg/cm^2^). With regard to segmental bioimpedance variables, the R, Xc, and PA were measured separately for each body segment, including the two arms, two legs, and trunk. As peripheral regions such as the arm and leg have appreciable variances, whereas the vital central organs are strongly resisted by thermoregulatory mechanisms (35), the segmental variables for the body trunk were ignored in the analysis. Using these extremity bioimpedance variables, we computed the upper (lower) extremities variables by averaging the corresponding variables in the right and left arms (legs) as suggested in our previous publication (31). In this study, two R ratio variables for the upper and lower extremities (i.e., Ri_Re_upper and Ri_Re_lower) were computed such that the R ratio was the difference between the R at 250 kHz and that at 5 kHz divided by the R at 5 kHz (Supplementary Table 1) (26, 36). Xc (Xc_upper, Xc_lower) and PA (PA_upper, PA_lower) variables in the upper and lower extremities were selected at 50 kHz of frequency. All measurements were performed in the supine position by well-trained staff who followed the instructions in the InBody S10 Manual (InBody S10, South Korea).

### Cold heat pattern questionnaire for the cold pattern

A list of eight questions related to cold syndromes was utilized to recognize CP and non-CP as described in Table 1. These eight questions were derived from the cold heat pattern questionnaire for the classification of cold group and the details can be found here (37). Each question has the lowest score of one and the highest score of five; hence, a maximum of forty points can be obtained. The cutoff score of 21.5 accurately predicted people with CP from non-CP participants with an area under the curve of 0.93 (sensitivity = 0.94, specificity = 0.80) and resulted in an agreement with experts’ diagnosis of up to 87.1% (38). Thus, this cutoff value was used as the threshold to classify individuals into either CP or non-CP groups.

**TABLE 1:** Description of the cold heat pattern questionnaire for cold pattern which was used in this study.

	Cold heat pattern questionnaire for cold pattern	Rating score
1	Aversion to cold	1–Incorrect
2	Preference for warmth	2–Somewhat
3	Abdominal coldness	incorrect
4	Coldness of the limbs	3–Normal
5	Coldness of the body	4–Somewhat
6	Pale face	correct
7	Usually drink warm water	5–Totally
8	Long voiding of colorless urine	correct
Score	Range: 8–40 8–21: Recognized as non-cold pattern 22–40: Recognized as cold pattern	

### Covariates

Several factors were considered as covariates in the multivariate regression analysis model in this study. First, age can influence the change in body composition (39) and the thermoregulatory responses (40–42). For instance, previous studies reported a relation between aging and body composition changes such as increasing body FM in tandem with decreasing lean mass and bone mineral density in older adults (43–45). Furthermore, older adults are unlikely to sense and respond to thermal imbalance appropriately compared to younger individuals (46). Therefore, age was included as a potential covariate in the logistic regression models. Second, previous studies reported significant relationship between educational levels and the changes in body composition, such that highly educated people tend to have lower BMI and FM compared to those with lower level of education (47, 48). To minimize the influence of educational levels since it shows significant difference between CP and non-CP groups, educational years was added as another covariate. Moreover, comorbidities that were significantly different between the two groups were included as additional covariates in the adjustment model and comprise systolic blood pressure, diastolic blood pressure, and osteoarthritis. In the second adjusted model in section “Relationship between the selected bioelectrical impedance analysis variables and cold patterns in women and men,” we considered height and weight as two additional covariates to lessen the difference between the body sizes when examining the associations between CP and the whole-body composition and segmental bioimpedance variables.

### Statistical analysis

Information on the participants’ demographics and body composition parameters are summarized as the mean and standard deviation (SD) and median and range (from minimum to maximum values) for continuous variables, and as frequency and proportions for categorical variables in the CP and non-CP groups. As women are more susceptible to CP (49) and have relatively higher FM in tandem with lower FFM than men (50), this study examined the changes in the body composition and impedance variables in association with the CP for women and men separately. A univariate independent two-sample *t*-test was used to compare the continuous variables between the two groups and Pearson’s chi-square test or Fisher’s exact test was used to check the independence of each categorical variable with CP and non-CP. Cohen’s d test was employed to examine the effect size of differences of each continuous variable between CP and non-CP groups.

Univariate and multivariate regression analyses were applied to investigate the association between CP and body composition variables using the estimated ORs and its 95% CI. Three models were constructed before and after controlling for covariates, including the crude model, first adjusted model, and the second adjusted model. In the crude model, no covariate was adjusted for. Age, systolic blood pressure, diastolic blood pressure, educational years, and osteoarthritis were included as covariates in the first adjusted model. The second adjusted model controlled for those in the first adjusted model, as well as height and weight. All of the continuous parameters were standardized to a mean of zero and a standard deviation (SD) of one before applying these regression analyses. Finally, a two-sample z-test was used to compare these associations between women and men. A *p*-value of less than 0.05 was considered statistically significant. R version 4.1.2 (The R Project for Statistical Computing; available at: http://www.r-project.org/) was used for all statistical analyses.

## Results

### Participant characteristics

Table 2 describes participants’ demographic characteristics, anthropometric measurements, and relevant comorbidities. There was a greater proportion of women in the CP group [290/(290+198) = 59.4%] than in the non-CP group [77/(77+102) = 44.3%]. In women, the systolic and diastolic blood pressures were significantly lower in the CP group than in the non-CP group. Among men, the CP group had a higher proportion of osteoarthritis and more years of education than those in the non-CP group. There was no difference in the mean age between the two groups for both women and men. In terms of the anthropometric measurements, with the exception of height, both CP women and men exhibited similar results of significantly lower BMI and less weight. Interestingly, the CP participants exhibited a significantly lower body volume per unit surface area compared to those with non-CP, with mean differences of approximately 1.1 L/m^2^ in both women and men, respectively.

**TABLE 2:** Demographic information, anthropometric measurements, and the relevant comorbidities in women and men.

	Women				Men			
Variables	Total (*n* = 367)^a^	CP (*n* = 290)^a^	Non-CP (*n* = 77)^a^	*p*-value^b^	Total (*n* = 300)^a^	CP (*n* = 198)^a^	Non-CP (*n* = 102)^a^	*p*-value^b^
**Age (year)**				0.393				0.206
Mean (SD)	71.0 (6.0)	71.2 (5.8)	70.5 (6.5)		73.1 (6.2)	73.4 (5.9)	72.5 (6.6)	
Median (range)	71.0 (56.0–86.0)	71.0 (58.0–86.0)	71.0 (56.0–85.0)		73.0 (56.0–89.0)	73.0 (56.0–87.0)	73.0 (57.0–89.0)	
**Education level (year)**				0.213				**0.011**
Mean (SD)	10.5 (4.2)	10.4 (4.2)	11.1 (4.1)		13.6 (4.7)	14.1 (4.6)	12.7 (4.9)	
Median (range)	11.0 (0.5–24.0)	10.5 (0.5–24.0)	11.0 (1.0–20.0)		14.0 (0.5–24.0)	14.0 (0.5–24.0)	14.0 (0.5–24.0)	
**Systolic blood pressure (mmHg)**				**0.002**				0.078
Mean (SD)	125.2 (14.7)	124.0 (14.8)	129.6 (13.4)		124.4 (13.9)	123.4 (14.0)	126.4 (13.6)	
Median (range)	125.0 (86.0–183.0)	124.0 (86.0–183.0)	131.0 (91.0–163.0)		124.0 (94.0–172.0)	123.0 (95.0–163.0)	125.5 (94.0–172.0)	
**Diastolic blood pressure (mmHg)**				**0.004**				0.427
Mean (SD)	71.9 (9.5)	71.2 (9.4)	74.7 (9.3)		69.5 (9.5)	69.2 (9.5)	70.1 (9.5)	
Median (range)	72.0 (47.0–98.0)	71.0 (50.0–98.0)	74.0 (47.0–96.0)		69.0 (36.0–99.0)	69.0 (47.0–99.0)	69.5 (36.0–96.0)	
**Height**				**0.041**				0.375
Mean (SD)	153.5 (5.3)	153.2 (5.3)	154.6 (5.5)		166.2 (5.5)	166.0 (5.6)	166.6 (5.3)	
Median (range)	153.2 (140.8–170.2)	152.8 (140.8–170.2)	154.4 (143.7–167.1)		165.6 (148.7–182.5)	165.6 (148.7–180.8)	165.9 (155.4–182.5)	
**Weight**				**<0.001**				**<0.001**
Mean (SD)	58.6 (7.4)	57.9 (7.3)	61.4 (7.1)		68.1 (8.7)	66.9 (9.3)	70.4 (7.1)	
Median (range)	58.0 (39.0–88.0)	57.4 (39.0–88.0)	60.9 (47.2–79.9)		68.1 (44.5–87.8)	67.0 (44.5–87.8)	70.4 (53.3–86.2)	
**BMI**				**0.004**				**<0.001**
Mean (SD)	24.9 (3.0)	24.7 (2.9)	25.7 (3.1)		24.6 (2.6)	24.2 (2.8)	25.4 (2.1)	
Median (range)	24.7 (17.7–35.8)	24.4 (17.7–35.8)	25.9 (19.7–34.8)		24.8 (17.6–31.2)	24.4 (17.6–31.2)	25.4 (20.5–29.9)	
**Volume-to-body surface area (liter/m^2^)**				<0.001				<0.001
Mean (SD)	36.0 (2.5)	35.7 (2.4)	36.8 (2.4)		36.4 (2.3)	36.0 (2.5)	37.1 (1.8)	
Median (range)	35.8 (27.0–44.6)	35.6 (27.0–44.6)	36.9 (31.9–42.8)		36.6 (29.8–41.5)	36.2 (29.8–41.5)	37.2 (32.3–40.9)	
**Diabetes, n (%)**				0.493				0.995
Yes	53 (14.4%)	40 (13.8%)	13 (16.9%)		53 (17.7%)	35 (17.7%)	18 (17.6%)	
No	314 (85.6%)	250 (86.2%)	64 (83.1%)		247 (82.3%)	163 (82.3%)	84 (82.4%)	
**Hypertension, n (%)**				0.064				0.495
Yes	152 (41.4%)	113 (39.0%)	39 (50.6%)		133 (44.3%)	85 (42.9%)	48 (47.1%)	
No	215 (58.6%)	177 (61.0%)	38 (49.4%)		167 (55.7%)	113 (57.1%)	54 (52.9%)	
**Hyperlipidemia, n (%)**				0.495				0.800
Yes	103 (28.1%)	79 (27.2%)	24 (31.2%)		42 (14.0%)	27 (13.6%)	15 (14.7%)	
No	264 (71.9%)	211 (72.8%)	53 (68.8%)		258 (86.0%)	171 (86.4%)	87 (85.3%)	
**Thyroid, n (%)**				0.753				0.341
Yes	16 (4.4%)	12 (4.1%)	4 (5.2%)		5 (1.7%)	2 (1.0%)	3 (2.9%)	
No	351 (95.6%)	278 (95.9%)	73 (94.8%)		295 (98.3%)	196 (99.0%)	99 (97.1%)	
**Osteoarthritis, n (%)**				0.542				**0.047**
Yes	159 (43.3%)	128 (44.1%)	31 (40.3%)		57 (19.0%)	44 (22.2%)	13 (12.7%)	
No	208 (56.7%)	162 (55.9%)	46 (59.7%)		243 (81.0%)	154 (77.8%)	89 (87.3%)	
**Alcohol twice a month, n (%)**				0.763				0.196
Yes	17 (4.6%)	13 (4.5%)	4 (5.2%)		100 (33.3%)	61 (30.8%)	39 (38.2%)	
No	350 (95.4%)	277 (95.5%)	73 (94.8%)		200 (66.7%)	137 (69.2%)	63 (61.8%)	
**Smoking habit, n (%)**				0.376				0.787
Yes	2 (0.5%)	1 (0.3%)	1 (1.3%)		19 (6.3%)	12 (6.1%)	7 (6.9%)	
No	365 (99.5%)	289 (99.7%)	76 (98.7%)		281 (93.7%)	186 (93.9%)	95 (93.1%)	

^a^The values represent mean (SD) for continuous variables, and n (%) for categorical variables.

^b^The *p*-values for the continuous variables were obtained from an independent two sample *t*-test. For the categorical variables, the *p*-values were derived from the chi-squared test statistics or Fisher-exact test. The *p*-value of lower than 0.05 were presented in boldface.

### Descriptions of the selected bioelectrical impedance analysis variables in women and men

**FIGURE 3:**
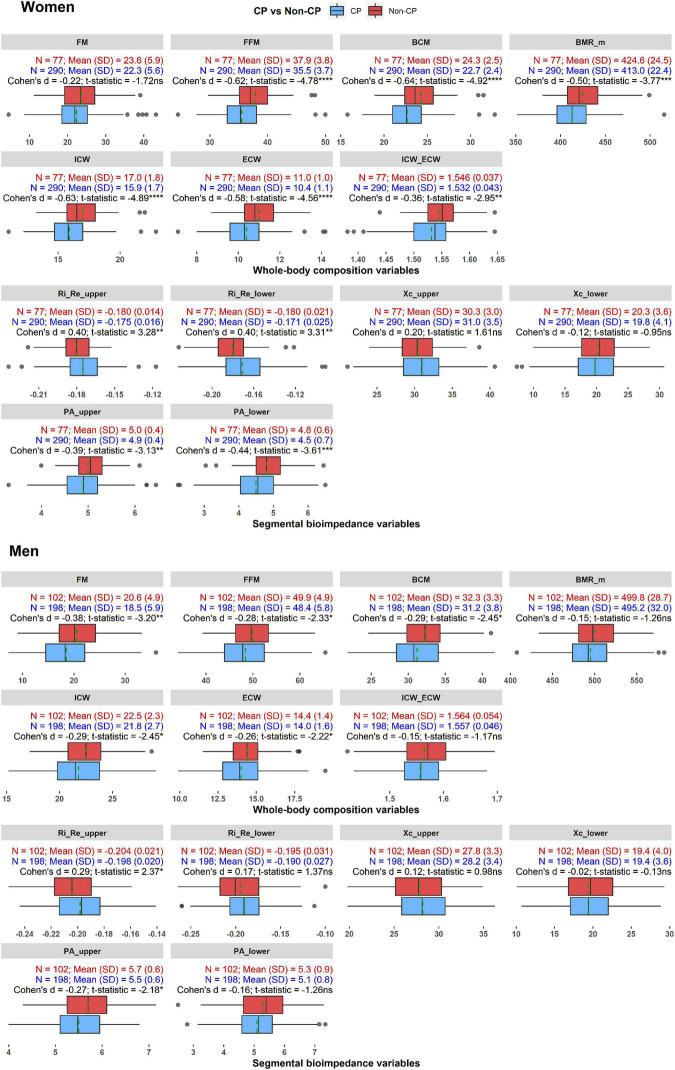
The distributions and comparisons of whole-body composition and segmental bioimpedance variables in the CP and non-CP groups in women and men. *p < 0.05, **p < 0.01, ***p < 0.001, ****p < 0.0001, ns: not significant. Dashed green line indicates mean value of each variable in each group.

Figure 3 shows the distributions and comparisons of whole-body composition and segmental bioimpedance variables in the CP and non-CP groups in women and men. In women, for the body composition variables, the CP group demonstrated a significantly lower body lean mass as indicated by the lower values of FFM and BCM compared to those of the non-CP participants. Accordingly, the water volume in the extracellular and intracellular spaces was reduced in the CP group compared with that in the non-CP group. Interestingly, BMR_m was lower in the CP group than in the non-CP group. The differences were prominent in these variables as the effect sizes, indicated by Cohen’s d results, ranged from −0.50 to −0.64. Moreover, ICW_ECW was significantly lower in the CP group than in the non-CP group with a moderate difference of Cohen’s d of −0.36. FM did not significantly differ between the two groups. The abovementioned patterns were similar in women and men except with BMR_m, ICW_ECW, and FM. In men, the effect sizes in FFM, BCM, ICW, and ECW were relatively smaller compared to those in women with Cohen’s d, ranging from −0.26 to −0.29. Nevertheless, men with CP did not show differences in BMR_m and ICW_ECW compared to those in the non-CP group, whereas FM was significantly lower in individuals with CP than in non-CP individuals (*d* = −0.38).

With regard to the segmental bioimpedance variables, R ratios and PAs in the upper or lower extremities differed significantly between women with and without CP. Particularly, PA_upper and PA_lower were lower (*d* = −0.39 and −0.44, respectively) while Ri_Re_upper and Ri_Re_lower were higher in the CP group compared to the non-CP group (*d* = 0.40). In men, only Ri_Re_upper and PA_upper differed significantly between the CP and non-CP groups (*d* = 0.29 and −0.27, respectively).

Comprehensive information on the associations depicted in Figure 2 is provided in Supplementary Table 2.

### Relationship between the selected bioelectrical impedance analysis variables and cold patterns in women and men

The associations between the selected BIA variables and CP were investigated separately in women and men (Table 3).

**TABLE 3:** Estimated odds ratios and their 95% confidence intervals derived from three logistic regression models in women and men.

Reference group: non-CP		Model 1: Crude			Model 2: Adjusted 1st			Model 3: Adjusted 2nd		
			
Group	Variables	OR^a^	95% CI^a^	*p*-value^b^	OR^a^	95% CI^a^	*p*-value^b^	OR^a^	95% CI^a^	*p*-value^b^
Women	FM	0.80	0.61, 1.03	0.086	0.84	0.64, 1.10	0.199	4.21	1.91, 9.63	**<0.001**
	FFM	0.28	0.16, 0.49	**<0.001**	0.28	0.15, 0.49	**<0.001**	0.14	0.05, 0.42	**<0.001**
	BCM	0.27	0.16, 0.47	**<0.001**	0.26	0.14, 0.46	**<0.001**	0.14	0.05, 0.38	**<0.001**
	BMR_m	0.36	0.20, 0.60	**<0.001**	0.32	0.18, 0.58	**<0.001**	0.22	0.09, 0.51	**<0.001**
	ICW	0.28	0.16, 0.47	**<0.001**	0.26	0.14, 0.46	**<0.001**	0.14	0.05, 0.39	**<0.001**
	ECW	0.31	0.18, 0.53	**<0.001**	0.32	0.18, 0.56	**<0.001**	0.21	0.07, 0.60	**0.004**
	ICW_ECW	0.67	0.49, 0.89	**0.006**	0.61	0.42, 0.86	**0.004**	0.59	0.41, 0.84	**0.003**
	Ri_Re_upper	1.72	1.21, 2.48	**0.003**	1.87	1.29, 2.76	**<0.001**	1.72	1.16, 2.57	**0.007**
	Ri_Re_lower	1.59	1.18, 2.18	**0.002**	1.75	1.23, 2.54	**0.002**	1.69	1.18, 2.48	**0.004**
	Xc_upper	1.21	0.92, 1.58	0.169	1.19	0.89, 1.59	0.243	1.08	0.80, 1.46	0.611
	Xc_lower	0.89	0.69, 1.14	0.353	0.88	0.66, 1.18	0.401	0.80	0.59, 1.07	0.134
	PA_upper	0.60	0.42, 0.84	**0.003**	0.56	0.38, 0.81	**0.002**	0.60	0.40, 0.89	**0.011**
	PA_lower	0.60	0.44, 0.82	**0.001**	0.55	0.38, 0.79	**<0.001**	0.57	0.39, 0.82	**0.002**
Men	FM	0.68	0.52, 0.88	**0.003**	0.69	0.52, 0.90	**0.005**	1.10	0.64, 1.91	0.724
	FFM	0.68	0.48, 0.96	**0.028**	0.64	0.42, 0.95	**0.025**	0.88	0.42, 1.82	0.724
	BCM	0.66	0.47, 0.94	**0.020**	0.61	0.40, 0.92	**0.019**	0.82	0.40, 1.67	0.590
	BMR_m	0.79	0.54, 1.15	0.222	0.76	0.48, 1.17	0.211	0.91	0.50, 1.67	0.763
	ICW	0.66	0.47, 0.94	**0.020**	0.61	0.40, 0.92	**0.019**	0.82	0.40, 1.67	0.587
	ECW	0.69	0.48, 0.98	**0.037**	0.66	0.44, 0.96	**0.031**	0.92	0.43, 1.97	0.827
	ICW_ECW	0.86	0.68, 1.09	0.219	0.91	0.68, 1.22	0.537	0.91	0.67, 1.23	0.542
	Ri_Re_upper	1.37	1.06, 1.78	**0.016**	1.28	0.95, 1.73	0.099	1.20	0.87, 1.65	0.265
	Ri_Re_lower	1.19	0.94, 1.52	0.151	1.17	0.87, 1.59	0.296	1.10	0.80, 1.52	0.545
	Xc_upper	1.14	0.88, 1.49	0.328	1.33	1.00, 1.79	0.051	1.17	0.86, 1.60	0.327
	Xc_lower	0.98	0.76, 1.26	0.894	1.13	0.84, 1.53	0.429	0.99	0.72, 1.37	0.953
	PA_upper	0.75	0.58, 0.97	**0.026**	0.81	0.60, 1.09	0.161	0.87	0.63, 1.20	0.399
	PA_lower	0.85	0.67, 1.08	0.186	0.88	0.65, 1.18	0.390	0.93	0.68, 1.28	0.673

^a^OR, odds ratio; CI, confidence interval.

^b^*p*-value obtains from Wald test. In both women and men, the second model adjusted for age, systolic blood pressure, diastolic blood pressure, education years, and osteoarthritis. The third model adjusted for those in the second model and height and weight. The *p*-value of lower than 0.05 were presented in boldface.

In women, regarding the whole-body composition variables, FFM, BCM, BMR_m, ICW, ECW, and ICW_ECW exhibited negative associations with CP before the covariates were controlled for, with OR (95% CI) ranging from 0.27 to 0.67 (0.16–0.89). One unit decrease in one of these variables corresponded to 33–73% increase the odds of CP. After adjusting for age and comorbidities, these associations remained significant at approximately the same magnitudes. Additionally, when including height and weight in the third model, the associated magnitudes achieved even greater values of OR (95% CI) and range from 0.14 to 0.59 (0.05–0.84). Furthermore, FM, which was not significantly associated with CP in the crude and first adjusted models, was positively associated with CP with OR (95% CI) of 4.21 (1.91–9.63). Among the segmental bioimpedance variables, the R ratios and PAs in the upper and lower extremities were significantly associated with CP in all three models, regardless of age, height, weight, and the other covariates. Particularly, in the last model, PA_upper and PA_lower produced negative associations with CP, with ORs (95% CI) of 0.60 (0.40–0.89) and 0.57 (0.39–0.82), Ri_Re_upper and Ri_Re_lower yielded positive associations with CP, with ORs (95% CI) of 1.72 (1.16–2.57) and 1.69 (1.18–2.48), respectively.

In men, before adjusting for the covariates, FM, FFM, BCM, ICW, and ECW showed approximately similar negative associations with CP, with ORs (95% CI) ranging from 0.66 to 0.69 (0.47–0.98). One unit decrease in one of these variables corresponded to 31–34% increase the odds of having CP. When taking age and other covariates into account in the second model, these associations remained significant at a slightly greater magnitude of OR. However, none of them showed a statistically significant association with CP after incorporating height and weight in the last model. For the segmental bioimpedance variables, only Ri_Re_upper and PA_upper were shown to be linked to CP in the crude model. After controlling for covariates in the second and the last model, all the segmental variables failed to produce an association with CP.

### Comparison of these associations between women and men

Differences in the associations with CP were reported between women and men (Table 4). Among the whole-body composition variables, except for ICW_ECW, all showed significantly stronger associations with CP in women than in men. Specifically, one unit increase in FM corresponded with a 282% increased odd of having CP in women with respect to men; whereas one unit decrease in either FFM, BCM, BMR_m, ICW, or ECW corresponded with 76–84% increased odd of having CP in women with respect to men. Most of segmental bioimpedance variables showed insignificant differences between women and men in their associations with CP.

**TABLE 4:** Odds ratios comparisons between women and men.

Variables	Δβ	e^Δβ^	95% CI	*Z*-score	*P*-value
FM	1.34	3.82	0.37, 2.31	2.70	**0.007**
FFM	–1.81	0.16	−3.12, −0.50	–2.70	**0.007**
BCM	–1.80	0.16	−3.08, −0.53	–2.77	**0.006**
BMR_m	–1.43	0.24	−2.48, −0.38	–2.67	**0.008**
ICW	–1.78	0.17	−3.06, −0.51	–2.74	**0.006**
ECW	–1.49	0.22	−2.81, −0.17	–2.22	**0.027**
ICW_ECW	–0.43	0.65	−0.91, 0.04	–1.79	0.073
Ri_Re_upper	0.36	1.44	−0.15, 0.87	1.39	0.163
Ri_Re_lower	0.43	1.54	−0.06, 0.92	1.72	0.085
Xc_upper	–0.08	0.93	−0.51, 0.36	–0.35	0.725
Xc_lower	–0.22	0.80	−0.66, 0.22	–0.97	0.330
PA_upper	–0.37	0.69	−0.88, 0.14	–1.42	0.156
PA_lower	–0.50	0.61	−0.99, −0.01	–2.01	**0.045**

Δβ, beta difference; e^Δβ^, exponential of beta difference; CI, confidence interval. The comparisons were made using the model which adjusted for age, education years, systolic blood pressure, diastolic blood pressure, osteoarthritis, height, and weight in both women and men. The *p*-value of lower than 0.05 were presented in boldface.

## Discussion

In this study, seven whole-body composition variables including FM, FFM, BCM, BMR_m, ICW, ECW, and ICW_ECW and three pairs of segmental bioimpedance variables consisting of R ratios, Xc results, and PAs in the upper and lower extremities were used to examine the associations with CP. Regarding the whole-body composition variables, we found that individuals with CP had significantly smaller body sizes, lesser body lean mass and water volume as expressed by a decrease in weight, FFM, BCM, ICW, and ECW than those with non-CP in both women and men. With respect to the segmental bioimpedance variables, we found significant increase in R ratios and decrease in PAs in the CP group compared with the non-CP group. After controlling for age, height, weight and the other covariates, these variables continued to significantly associate with the CP prevalence, especially in women. From previous studies, some of these variables have been investigated in people with cold hypersensitivity symptoms. Particularly, they reported significantly lower body lean mass indices, FM indices, or ICW_ECW ratios in the cold group when compared with the non-cold or heat group (11, 19, 21). Additionally, whole-body PA was found to be negatively associated with the incidence of CP (11). Our findings are consistent with these results.

First of all, in terms of the whole-body composition variables, we found significant reductions in BMI, FFM, and BCM in both women and men. In women, the lower BMI might be due to a decrease in FFM or BCM mainly, rather than a decline in FM as indicated by the significantly lower FFM and BCM and indifferent FM when compared with those of the non-CP participants (Figure 3). After controlling for age, height, weight, and the comorbidities, FFM and BCM exhibited significantly negative associations with the CP prevalence. Interestingly, FM, which was not associated with CP in the crude model, became positively associated with CP in the last model (Table 3). In men, BMI reduction may be caused by a decrease in both FFM and FM as demonstrated by the relatively lower FFM and FM in the CP group (Figure 3). However, the reductions in these lean and FM variables might be subtle; therefore, they failed to produce significant associations with CP after controlling for age, height, weight, and other covariates.

The abovementioned observations between lower body lean mass and CP prevalence might be explained by the human thermoregulation and thermal equilibrium mechanisms. Evidently, our human body generates heat internally and maintains a core temperature of approximately 37°C, regardless of the environment (51). The heat generations are mainly functions of healthy cellular mitochondria in the body lean mass (52); thus, individuals with lower body lean mass may have relatively lower amount of heat production. On the contrary, FM tissue has been considered an insulation layer to protect body temperature expenditure (53, 54). Lower FFM and BCM together with a higher FM suggest that the heat production might be insufficient to compensate for the heat loss despite a thicker insulative layer, as shown in our CP individuals. Consequently, CP individuals are more sensitive to a given thermal stimulus (e.g., cold wind) whereby the nervous system is activated and enhances peripheral vasoconstriction to protect the core temperature (55). Therefore, CP individuals with low body lean mass tend to feel aversion to cold, especially on their extremities, where the fluctuation of temperature is more perceptible than in the body trunk. Moreover, when heat production is insufficient, the body trunk temperature might be deficient; therefore, these individuals may also experience abdominal discomfort with cold. Subsequently, individuals with CP are likely to have lower body lean mass and be more susceptible to ambient thermal fluctuations than those among the non-CP individuals, as shown in our studies with women regardless of age, height, and weight.

In addition, BMR which is highly correlated with the body lean mass variables was also decreased in the CP group (56, 57). In this study, we calculated BMR with respect to the body weight (BMR_m) as proposed by a previous report (32). Physiologically, BMR_m reflects the actual rate of metabolism during heat production (56) such that the higher the BMR_m, the more the heat that will be generated. Our result of BMR_m reduction confirmed the inferiority of the heat production in individuals with CP. However, since human body produces heat mainly from healthy cells in lean mass such as the brain, liver, or skeletal muscle mass, but hardly from the adipose tissue (58, 59), future studies with a mass-specific BMR might provide more insight into how different body masses manipulate BMR changes in individuals with CP.

Another variable was included to investigate the changes in body volume in relation to the body surface area, named as the volume-to-body surface area, between the CP and non-CP groups. From the above discussions, the CP individuals have a smaller body size as expressed by the lower weight and height and lesser body lean mass; thus, they produce less heat compared to the non-CP individuals. As revealed by the significantly lower body volume per surface area, our results suggested that the CP individuals not only have a smaller body size but also a relatively higher body surface area compared to the non-CP individuals. According to Bergmann’s rule, individuals with small body volume and higher body surface area are unlikely to be able to withstand cold temperature due to the imbalance between the lesser heat production and greater heat loss (33, 60). The findings of this study are consistent with the Bergmann’s rule.

Based on the water-related variables, we found that individuals with CP had a reduced total body water volume as indicated by the decrease in both ICW and ECW. ICW reflects the BCM, whereas, ECW is associated with the nutritional status of the individual (61, 62). Nevertheless, the decrease in body lean mass might cause the reduction in ICW and ECW. Moreover, we found an abnormal cellular water distribution as demonstrated by the decrease in ICW_ECW ratio in CP individuals compared to the non-CP individuals. ICW_ECW has been considered a prognostic factor of several pathological conditions such as edema, sarcopenia, cancer, and cognitive decline, and is associated with muscle strength and gait speed in older populations (27, 63–66). Decreased ICW_ECW suggests more serious reduction in the intracellular area water compared to that in the extracellular space and might have been caused by the lower BCM, body cell volume, or a malnutrition status in the CP individuals.

Regarding the segmental bioimpedance variables, the R ratios and PAs in the upper and lower extremities differed significantly between the CP and non-CP groups, especially in women, after controlling for the covariates; however, the results were not significant in men. The R ratios were calculated to reflect the changes in ICW/ECW between the upper and lower extremities, such that its positive association with the CP incidence suggested a decrease in ICW compared to ECW (26, 36). This finding is in line with the result of whole-body ICW/ECW ratio described above. The OR results indicated comparable magnitudes of changes of the abnormal cellular water distributions in the upper and lower extremities. Furthermore, the PAs in the upper and lower extremities were negatively associated with the incidence of CP. PA has been considered a useful indicator of BCM and cell membrane integrity in many clinical situations (23, 67, 68) and in relation with the hydration status of the body (69, 70). Marini and colleagues reported a positive correlation between PA and ICW/ECW ratio (or negatively correlated with ECW/ICW ratio) in that the water volume was measured by the dilution technique which is the gold standard to access body fluid (69). In addition, PA appeared to link with the body lean mass quantities such as FFM, FFM index, or skeletal muscle mass (70, 71). Since the majority of FFM is ICW (72), PA also positively correlates with ICW rather than with ECW, especially in men (69). Thus the decrease of PAs corresponds with the lower ICW or ICW with respect to ECW (ICW/ECW), and is associated with the reduction of body lean mass. These aforementioned variables are highly correlated to each other (73). In this relationship, we found that both the whole-body lean mass quantities, ICW/ECW ratio, and segmental PAs exhibited similar negative associations with CP prevalence. Our findings were consistent with these results and indicated a lower BCM quantitatively, or an impairment of the body cell membrane capacitance and integrity in the CP group compared to the non-CP individuals. Notably, after considering BCM as an additional covariate, these associations between segmental bioimpedance variables and CP prevalence diminished (Supplementary Table 3).

From the perspective of sex differences, the associations obtained from the whole-body composition and segmental bioimpedance variables with CP prevalence differed significantly between women and men. Previous studies have suggested that women have a lower metabolic heat production and greater insulative response during cold stress experiences than men (74). A smaller body lean mass and higher burden of FM might be the cause for these observations. Compared to men, women have relatively lower surface area and larger peripheral heat sink; hence, they are less capable of acclimating with heat (75). Furthermore, despite having a greater insulation layer from body FM, women maintain a constant rectal temperature at a greater metabolic cost than men (76). Furthermore, in terms of thermal sensations, women require a higher thermal comfort zone and tend to experience more discomfort than men when exposed to cooling situations, especially in their extremities (77, 78). For these reasons, women may be more susceptible with CP, thus they presented more pronounced associations with CP than the men.

According to age, thermal sensations in relation to CP susceptibility vary across different age groups (79). A common agreement is that older adults are more susceptible to cold hypersensitivity than younger people (40, 41). Older adults have relatively low heat production and a narrow thermoneutral zone that may be due to impaired thermal perception and weakened autonomic and behavioral thermoregulatory responses (41, 42). Compared to younger individuals, older individuals are unlikely to sense and respond to a thermal imbalance appropriately (46). Thus, physiological aging can influence the probability of having CP in older people. In this study, the impact of age as well as comorbidities seemed negligible, which might be due to the statistical indifference in the mean age between groups.

Women in the CP group had slightly lower systolic and diastolic blood pressure than those in the non-CP group. Similarly, although the difference did not reach the significant threshold of 0.05, men with CP exhibited a trend of lower systolic blood pressure than those without CP. Previous studies reported a lower level of blood pressure in the individuals with cold extremities syndromes, especially in women (9, 80). They suggested a relationship between blood pressure and feeling of cold in the extremities, as in the Flammer syndrome. This syndrome explains a phenomenon of when people react to stimuli like cold by altering their blood pressure (80, 81). The mechanisms of the relationship between body weight and height with blood pressure are complex (82). Previous studies suggested that body weight and BMI have positive associations with systolic blood pressure whereas body height mainly affects the diastolic blood pressure in a negative correlative manner (83, 84). Nevertheless, the Flammer syndrome or the influence of body size and BMI might contribute to the lower blood pressures in the CP group in our study.

This study provided an insight into how whole-body composition and segmental bioimpedance change in relation to CP incidence for different sexes. The results suggest that individuals with abnormal cellular water distribution, insufficient BCM and/or body cell strength might be susceptible to cold hypersensitivity symptoms. An increase in BCM or overall health status induced by a well-balanced diet and regular physical activities might help protect the body from CP.

In this study, the prevalence of CP was approximately 73% (448/667), which is higher than the 60% proportion of cold hypersensitivity in the general population in Korea or Japan (3, 4). The selection of participants of older age (55 years of age and above) who are more susceptible to cold hypersensitivity compared to the younger people (40–42) may be one of the reasons for this higher prevalence of CP. Subsequently, younger CP participants may show different relationships between body composition and bioimpedance variables due to their different physiological and pathological conditions. Additionally, the CP questionnaire that was adopted in this study is simple, with only eight questions, and the accuracy might have been improved if a longer questionnaire was used (37, 38). Diagnosing CP using objective criteria can aid in the generalization of these findings.

## Conclusion

This study investigated the association between CP and seven whole-body compositions with three pairs of segmental bioimpedance variables. Lean mass was significantly lower, whereas FM was relatively higher in individuals with CP than in those without CP, regardless of age, height, weight, and other covariates. Correspondingly, body volume with respect to the surface area and BMR_m were lower in the CP group than in the non-CP group. Segmental bioimpedance analysis revealed that individuals with CP have abnormal cellular water distributions as well as a significantly lower BCM and/or cell membrane strength in both upper and lower extremities. These findings emerged only in women but not in men. The behaviors in these body lean and segmental bioimpedance variables may be considered as potential markers for identifying CP, especially in women.

## Data availability statement

The original contributions presented in the study are included in the article/Supplementary material, further inquiries can be directed to the corresponding author.

## Ethics statement

The studies involving human participants were reviewed and approved by the Institutional Review Board of Chonnam National University Hospital (approval number: CNUH-2019-279). The patients/participants provided their written informed consent to participate in this study.

## Author contributions

DD analyzed the data and wrote the manuscript. KK handled the Institutional Review Board approval and managed the data. SK assisted in the data analysis. SL developed and validated the cold pattern identification questionnaire. KL took care of the data collection and curation. JK designed the study and wrote the manuscript. All authors revised and approved the contents of the manuscript, contributed to the article, and approved the submitted version.
