# Supplementary material for: Segmental bioelectrical impedance analysis for Korean older population with cold pattern
Source: Front Nutr. 2022 Dec 1;9:975464. doi: 10.3389/fnut.2022.975464 (PMC9751898; doi:10.3389/fnut.2022.975464)
Supplement: Supplementary file 1 [file Data_Sheet_1.docx]

Supplementary Material

**Table S1**. Description of the selected variables considered in this study

| **Variable** | **Description** | **Unit** | **Definition** | |
| --- | --- | --- | --- | --- |
| BMR_m | Basal metabolic rate per unit mass | kJ/kg.d | BMR_m = $\frac{BMR}{{Weight(kg)}^{0.25}}$ | |
| Ri_Re_upper | Ratio of resistance difference between those at 250 kHz and those at 5 kHz and resistance at 5 kHz in the upper extremities. [Jaffrin MY and Morel H. 2008, Yamada Y et al., 2017] | - | ($\frac{R_{\mathrm{RA}_{250kHz}}-R_{\mathrm{RA}_{5kHz}}}{R_{\mathrm{RA}_{5kHz}}}$ + $\frac{R_{\mathrm{LA}_{250kHz}}-R_{\mathrm{LA}_{5kHz}}}{R_{\mathrm{LA}_{5kHz}}})/2$ | |
| Ri_Re_lower | Ratio of resistance difference between those at 250 kHz and those at 5 kHz and resistance at 5 kHz in the lower extremities. | - | ($\frac{R_{\mathrm{RL}_{250kHz}}-R_{\mathrm{RL}_{5kHz}}}{R_{\mathrm{RL}_{5kHz}}}$ + $\frac{R_{\mathrm{LL}_{250kHz}}-R_{\mathrm{LL}_{5kHz}}}{R_{\mathrm{LL}_{5kHz}}})/2$ | |
| Xc_upper | Average of segmental reactance in upper limbs at 50 kHz | Ohm | $\frac{\mathrm{Xc}_{\mathrm{RA}}+\mathrm{Xc}_{\mathrm{LA}}}{2}$ | |
| Xc_lower | Average of segmental reactance in lower limbs at 50 kHz | Ohm | $\frac{\mathrm{Xc}_{\mathrm{RL}}+\mathrm{Xc}_{\mathrm{LL}}}{2}$ | |
| PA | Phase angle at 50 kHz | Degree | tan^-1^ ($\frac{\mathrm{Xc}}{sqrt(Z^2-Xc^2)})*180/\pi$ | |
| PA_upper | Average of segmental phase angle in upper limbs at 50 kHz | Degree | $\frac{\mathrm{PA}_{\mathrm{RA}}+\mathrm{PA}_{\mathrm{LA}}}{2}$ | |
| PA_lower | Average of segmental phase angle in lower limbs at 50 kHz | Degree | $\frac{\mathrm{PA}_{\mathrm{RL}}+\mathrm{PA}_{\mathrm{LL}}}{2}$ | |
| RA: Right arm, LA: Left arm, RL: Right leg, LL: Left leg, Z: Impedance, Xc: Reactance, R: Resistance, PA: Phase angle – These parameters were directly derived from Inbody S10. | | | |  |

**Table S2.** Descriptions of figure 2 - whole-body composition and segmental bioimpedance variables in women and men with CP and non-CP

|  | **Women** | | | | **Men** | | | |
| --- | --- | --- | --- | --- | --- | --- | --- | --- |
| **Variables** | **Total (n = 367)^a^** | **CP (n=290)^a^** | **Non-CP (n=77)^a^** | ***p*_value^b^** | **Total (n = 300)^a^** | **CP (n=198)^a^** | **Non-CP (n=102)^a^** | ***p*_value^b^** |
| **FM** |  |  |  | 0.078 |  |  |  | **0.002** |
| Mean (SD) | 22.6 (5.6) | 22.3 (5.5) | 23.6 (5.9) |  | 19.2 (5.7) | 18.5 (5.9) | 20.6 (4.9) |  |
| Median [range] | 22.2 [8.6-43.2] | 21.9 [8.6-43.2] | 23.4 [11.1-39.1] |  | 19.1 [7.3-36.0] | 18.3 [7.3-36.0] | 20.1 [9.0-33.3] |  |
| **FFM** |  |  |  | **<0.001** |  |  |  | **0.025** |
| Mean (SD) | 36.0 (3.8) | 35.6 (3.7) | 37.9 (3.8) |  | 48.9 (5.6) | 48.4 (5.8) | 49.9 (4.9) |  |
| Median [range] | 35.8 [24.5-50.0] | 35.3 [24.5-50.0] | 37.0 [29.7-48.2] |  | 48.7 [34.1-65.4] | 47.9 [34.1-65.4] | 49.6 [39.3-63.2] |  |
| **BCM** |  |  |  | **<0.001** |  |  |  | **0.016** |
| Mean (SD) | 23.07 (2.53) | 22.74 (2.44) | 24.31 (2.52) |  | 31.6 (3.7) | 31.2 (3.8) | 32.3 (3.3) |  |
| Median [range] | 22.90 [15.80-32.80] | 22.60 [15.80-32.80] | 23.60 [18.90-31.50] |  | 31.4 [21.8-41.7] | 30.8 [21.8-41.7] | 32.2 [24.6-41.2] |  |
| **BMR_m** |  |  |  | **<0.001** |  |  |  | 0.237 |
| Mean (SD) | 415 (23) | 413 (22) | 425 (25) |  | 497 (31) | 495 (32) | 500 (29) |  |
| Median [range] | 414 [351-516] | 413 [351-516] | 421 [380-499] |  | 495 [408-582] | 492 [408-582] | 498 [434-570] |  |
| **ICW** |  |  |  | **<0.001** |  |  |  | **0.016** |
| Mean (SD) | 16.11 (1.77) | 15.88 (1.70) | 16.97 (1.76) |  | 22.04 (2.56) | 21.80 (2.66) | 22.52 (2.29) |  |
| Median [range] | 16.00 [11.00-22.90] | 15.80 [11.00-22.90] | 16.50 [13.20-22.00] |  | 21.95 [15.20-29.10] | 21.50 [15.20-29.10] | 22.50 [17.20-28.70] |  |
| **ECW** |  |  |  | **<0.001** |  |  |  | **0.042** |
| Mean (SD) | 10.49 (1.08) | 10.36 (1.06) | 10.97 (1.04) |  | 14.13 (1.56) | 14.00 (1.64) | 14.40 (1.36) |  |
| Median [range] | 10.40 [7.10-14.20] | 10.30 [7.10-14.20] | 10.80 [8.70-13.50] |  | 14.10 [9.90-19.50] | 13.90 [9.90-19.50] | 14.40 [11.50-17.80] |  |
| **ICW_ECW** |  |  |  | **0.008** |  |  |  | 0.103 |
| Mean (SD) | 1.535 (0.043) | 1.532 (0.044) | 1.546 (0.037) |  | 1.559 (0.049) | 1.557 (0.046) | 1.564 (0.054) |  |
| Median [range] | 1.538 [1.381-1.646] | 1.538 [1.381-1.646] | 1.551 [1.439-1.646] |  | 1.564 [1.421-1.695] | 1.558 [1.433-1.681] | 1.571 [1.421-1.695] |  |
| **Ri_Re_upper** |  |  |  | **0.003** |  |  |  | **0.025** |
| Mean (SD) | -0.176 (0.016) | -0.174 (0.016) | -0.180 (0.014) |  | -0.200 (0.020) | -0.198 (0.020) | -0.204 (0.021) |  |
| Median [range] | -0.176 [-0.234--0.117] | -0.175 [-0.234--0.117] | -0.180 [-0.218--0.152] |  | -0.199 [-0.252--0.141] | -0.197 [-0.244--0.141] | -0.204 [-0.252--0.159] |  |
| **Ri_Re_lower** |  |  |  | **0.002** |  |  |  | **0.049** |
| Mean (SD) | -0.173 (0.024) | -0.171 (0.025) | -0.180 (0.021) |  | -0.192 (0.029) | -0.190 (0.027) | -0.195 (0.031) |  |
| Median [range] | -0.173 [-0.233--0.091] | -0.172 [-0.233--0.091] | -0.179 [-0.232--0.121] |  | -0.194 [-0.264--0.100] | -0.191 [-0.260--0.112] | -0.200 [-0.264--0.100] |  |
| **Xc_upper** |  |  |  | 0.168 |  |  |  | 0.340 |
| Mean (SD) | 30.8 (3.5) | 31.0 (3.6) | 30.3 (3.0) |  | 28.1 (3.3) | 28.2 (3.4) | 27.8 (3.3) |  |
| Median [range] | 30.8 [17.7-40.6] | 31.0 [17.7-40.6] | 30.4 [24.0-38.6] |  | 28.0 [19.8-36.2] | 28.1 [19.8-36.2] | 27.7 [19.8-34.9] |  |
| **Xc_lower** |  |  |  | 0.303 |  |  |  | 0.687 |
| Mean (SD) | 19.9 (4.0) | 19.8 (4.1) | 20.3 (3.6) |  | 19.4 (3.7) | 19.4 (3.6) | 19.4 (4.0) |  |
| Median [range] | 19.9 [7.4-30.8] | 19.8 [7.4-30.8] | 20.5 [10.0-28.5] |  | 19.6 [10.1-29.3] | 19.5 [10.7-28.9] | 19.7 [10.1-29.3] |  |
| **PA_upper** |  |  |  | **0.004** |  |  |  | **0.034** |
| Mean (SD) | 4.91 (0.45) | 4.88 (0.45) | 5.05 (0.40) |  | 5.57 (0.58) | 5.52 (0.56) | 5.67 (0.60) |  |
| Median [range] | 4.90 [3.30-6.45] | 4.90 [3.30-6.45] | 5.05 [4.00-6.10] |  | 5.55 [4.00-7.15] | 5.47 [4.00-6.80] | 5.70 [4.30-7.15] |  |
| **PA_lower** |  |  |  | **<0.001** |  |  |  | 0.066 |
| Mean (SD) | 4.59 (0.72) | 4.52 (0.74) | 4.82 (0.61) |  | 5.17 (0.85) | 5.12 (0.81) | 5.26 (0.92) |  |
| Median [range] | 4.60 [2.25-6.50] | 4.55 [2.25-6.50] | 4.80 [3.05-6.45] |  | 5.20 [2.50-7.35] | 5.15 [2.80-7.35] | 5.40 [2.50-7.30] |  |
| ^a^The values represent mean (SD) for continuous variables, and n (%) for categorical variables.  ^b^The p-values for the continuous variables were obtained from an independent two sample t-test. For the categorical variables, the p-values were derived from the chi-squared test statistics or Fisher-exact test. | | | | | | | | |

**Table S3.** Estimated odds ratios and 95% confidence intervals derived from the logistic regression model upon adding BCM as an additional covariate.

| **Reference group: non-CP** | | **Model 4: Adjusted 3nd** | | |
| --- | --- | --- | --- | --- |
| **Group** | **Variables** | **OR^a^** | **95% CI^a^** | ***p*-value^b^** |
| **Women** | **ICW_ECW** | 0.70 | 0.48, 1.00 | 0.053 |
|  | **Ri_Re_upper** | 1.46 | 0.97, 2.21 | 0.072 |
|  | **Ri_Re_lower** | 1.39 | 0.95, 2.05 | 0.092 |
|  | **Xc_upper** | 0.96 | 0.70, 1.31 | 0.781 |
|  | **Xc_lower** | 0.84 | 0.62, 1.14 | 0.261 |
|  | **PA_upper** | 0.71 | 0.47, 1.06 | 0.096 |
|  | **PA_lower** | 0.70 | 0.47, 1.03 | 0.068 |
| **Men** | **ICW_ECW** | 0.95 | 0.70, 1.28 | 0.738 |
|  | **Ri_Re_upper** | 1.20 | 0.89, 1.64 | 0.230 |
|  | **Ri_Re_lower** | 1.07 | 0.78, 1.47 | 0.666 |
|  | **Xc_upper** | 1.20 | 0.88, 1.65 | 0.244 |
|  | **Xc_lower** | 1.07 | 0.78, 1.46 | 0.678 |
|  | **PA_upper** | 0.86 | 0.63, 1.18 | 0.354 |
|  | **PA_lower** | 0.96 | 0.70, 1.31 | 0.809 |
| ^a^OR = Odds Ratio, CI = Confidence Interval | | | | |
| \| ^b^The *p*-value obtains from Wald test. Model 4 controlled for all the covariates in the second adjusted model and BCM. \| \| --- \| | | | | |
